# Quantitative PCR Detection and Characterisation of Human Adenovirus, Rotavirus and Hepatitis A Virus in Discharged Effluents of Two Wastewater Treatment Facilities in the Eastern Cape, South Africa

**DOI:** 10.1007/s12560-016-9246-4

**Published:** 2016-05-28

**Authors:** Martins Ajibade Adefisoye, Uchechukwu U. Nwodo, Ezekiel Green, Anthony Ifeanyin Okoh

**Affiliations:** 1 SAMRC Microbial Water Quality Monitoring Centre, University of Fort Hare, Alice, 5700 South Africa; 2 Applied and Environmental Microbiology Research Group, Department of Biochemistry and Microbiology, University of Fort Hare, Alice, 5700 South Africa

**Keywords:** Human adenovirus, Environmental microbiology, Wastewater, qPCR, Public health

## Abstract

The occurrence of enteric viruses in reclaimed wastewater, their removal by efficient treatment processes and the public health hazards associated with their release into the environments are of great significance in environmental microbiology. In this study, TaqMan-based real-time polymerase chain reaction (qPCR) was used to assess the prevalence of human adenovirus (HAdV), rotavirus (RV) and hepatitis A virus (HAV) in the final effluents of two wastewater treatment plants in the Eastern Cape Province, South Africa, over a twelve-month sampling period. The correlation between the concentrations of viruses in the effluents samples and faecal coliform (FC) densities were assessed as to validate the use of FC as microbiological indicator in water quality assessment. HAdV was detected in 62.5 % (30/48) of the samples with concentrations ranging between 8.4 × 10^1^ and 1.0 × 10^5^ genome copies/L while HAV and RV were only detected at concentrations below the set detection limits. FCs densities ranged from 1 to 2.7 × 10^4^ CFU/100 ml. Adenovirus species HAdV-B (serotype 2) and HAdV-F (serotype 41) were detected in 86.7 % (26/30) and 6.7 % (2/30) of the HAdV-positive samples, respectively. No consistent seasonal trend was observed in HAdV concentrations, however, increased concentrations of HAdV were generally observed in the winter months. Also, there was no correlation between the occurrence of HAdV and FC at both the treatment plants. The persistent occurrence of HAdV in the discharged treated effluents points to the potential public health risk through the release of HAdV into the receiving watersheds, and the possibility of their transmission to human population.

## Introduction

The prevalence of enteric viruses in aquatic environment varies widely and depends largely upon human activities (Eifan 2013). High concentrations of human enteric viruses are often shed in the faeces of infected individuals, and may find their ways into a variety of aquatic environment and food, particularly in areas with poor sanitary infrastructures (Sibanda and Okoh 2012). They can be transmitted from these sources back to susceptible individuals to continue the cycle of disease (Rzezutka and Cook 2004). Enteric viruses of public health importance include different groups of viruses present in the intestinal tracts of human and animals, and are associated with a wide array of illnesses in susceptible hosts.

Adenoviruses are non-enveloped, double-stranded DNA viruses with diameter ranging between 70 and 90 nm. Their viral capsid consists of 240 hexons and 12 penton bases, each with a fibre protruding from the viral particle surface, and giving them characteristic morphological appearance (Harrach et al. 2012). HAdVs have been linked to a wide range of community and institutional disease outbreaks and have been isolated from practically all human organ systems (Lu et al. 2014). They are considered one of the most important pathogenic viral agents of infantile gastroenteritis after rotavirus (Zlateva et al. 2005; Mans et al. 2010; Elhag et al. 2013). There are presently 69 recognised genotypes of HAdV. These are classified into seven species, designated species A to G based on physical, chemical and genetic properties, and new genotypes are being recognised using phylogenetic analysis based on complete genome sequencing (Harrach et al. 2012; Lu et al. 2014).

Hepatitis A virus (HAV) is a small non-enveloped virus belonging to the family *Picornaviridae*, a leading cause of acute viral hepatitis with annual estimated cases of about 1.5 million worldwide (Franco et al. 2012). The complete genomic characterisation of HAV through sequencing of the VP1/2A junction and the VP1 gene indicated 3 genotypes (I, II and III) which could be grouped into subtypes A and B (both have been described for human), while genotypes IV, V and VI have been described for other primates (Coudray-Meunier et al. 2014). Sanitation and socioeconomic status are two key factors defining the geographical distribution of HAV, while Africa, the Middle East, India, Central and South America remain endemic regions for HAV. It is mainly transmitted via the faecal-oral route through human-to-human contact or by ingesting contaminated water and food, such as shellfish, soft fruit and uncooked vegetable.

Similarly, rotavirus (RV) is an infectious virus that causes damage to the lining of the small intestine leading to gastroenteritis. RV is a double-stranded RNA virus belonging to the family *Reoviridae*. It is mainly transmitted through the faecal-oral route by ingesting contaminated food and fluid including water, airborne droplets and person-to-person contact. RV is the most common aetiological viral agent of severe diarrhoea among infants and young children in developed and developing countries (Sherchand et al. 2012; Al-Badani et al. 2014; Rao et al. 2015). Clinical presentation of rotavirus infection includes fever, vomiting and watery diarrhoea, severe dehydration and stomach pain that may last between 3 and 5 days. Based on the antigenicity and nucleotide sequence analysis of the VP6 gene, 8 different species of RV have been identified and designated as species A through to G (Kindler et al. 2013). RV is shed in large concentrations by infected individual, and like other enteric viruses, is commonly found in domestic wastewater and can contaminate surface water sources (Kiulia et al. 2010). It is known to exhibit greater resistance to common disinfectant agents than most other enteric viruses and can survive in environmental water for days to weeks depending on the quality of the water and its temperature (Xagoraraki et al. 2014).

Faecal contamination of environmental water may play an important role in the transmission and epidemiology of enteric viruses (Enriquez et al. 1995; Bosch 1998; Sdiri-Loulizi et al. 2010), especially if such water is directly used for recreational purpose or serve as source water for portable water treatment plants. HAdV, on the other hand, has the potential to survive in sewage more than other enteric viruses, and can remain infectious for a prolonged period of time in environmental waters (Carducci et al. 2009; Quidort 2013). They can resist disinfection, heating, pressure and low pH (Koopmans and Duizer 2004; Carducci and Verani 2013) hence; inadequate chlorination and reduced contact time may lead to failure in the removal of some viral pathogens during disinfection (Fong and Lipp 2005).

Despite advances in water/wastewater treatment technologies, nonetheless, waterborne outbreaks remain a significant threat to human health worldwide, especially, in developing countries where large portion of the populations still depend on untreated surface water for their immediate water needs (Amenu 2014). These surface waters are, in many instances, impacted adversely by inadequately treated wastewater effluents, due to lack or non-implementation of regulations regarding the microbiological quality of treated effluents (Tyagi et al. 2006).

Coliforms are still largely used as indicators of faecal contamination in water quality assessment. However, it is essential to note the lack of correlation that often occurs between the presence of bacterial and viral pathogens in water systems (He et al. 2011). Traditional water quality indicators such as faecal coliforms (FC) give little or no information on the presence and concentrations of enteric viruses in water systems (Eifan 2013).

Reclaimed wastewater is an important resource in South Africa; a country described as water-stressed due to dwindling rainfall and increasing freshwater degradation, which partly has been linked to indiscriminate discharge of untreated and inadequately treated municipal effluents into the scarce freshwater resource of the country (Gopo 2013). Presently, the microbiological quality of effluents is still largely assessed by means of faecal coliform in South Africa, while adequate information on the virological quality of discharged effluents is deficient. A number of studies have documented the virological qualities of some freshwater resources in the country (Grabow et al. 2004; van Zyl et al. 2006; Olaniran et al. 2012; Chigor and Okoh 2012) but similar information on the significance and epidemiological importance of enteric viruses from wastewater effluent discharges into the environment have not been fully elucidated. Moreover, environmental surveillance is important in a developing country like South Africa where appropriate system for outbreak reporting and surveillance is deficient. Considering the strategic position, wastewater treatment plants occupy as hotspots for the dissemination of enteric viruses, the highly infectious nature of viruses, and low infectious doses required for infection (Nadan et al. 2003; La Rosa et al. 2012; Murray et al. 2013), assessment of the virological quality of discharged wastewater effluents for enteric viruses of epidemiological importance becomes highly imperative. Quantitative real-time polymerase chain reaction (qPCR) has proven to be a sensitive and specific tool for viral nucleic acid detection and quantification, and has been a very useful and reliable method for microbial risk and assessment in food and water applications (Dunn et al. 2014). Therefore, this study was designed to utilise quantitative and qualitative PCR techniques to assess the prevalence of HAdV, HAV and RV in the discharged final effluents of two wastewater treatment facilities in the Eastern Cape Province of South Africa and to correlate the prevalence of the viruses to faecal indicator bacteria in the final effluents.

## Materials and Methods

### Design of the Study and Source of Samples

The two wastewater treatment plants studied are located in Eastern Cape Province, South Africa and named Stutterheim Wastewater Treatment Plants (SWTP) on geographical coordinates 32°34′17″S, 27°26′95″E and Keiskammahoek Wastewater Treatment Plants (KWTP) on coordinates 32°41′31″S, 27°08′36″E. Both treatment plants utilise the activated sludge technology, and discharge their final effluents into the Cumakala and Keiskamma rivers, respectively. Collection of wastewater final effluents samples was done monthly (September 2012 to August 2013) from both the chlorination final effluent tanks (FE) and the discharge points (DP) of the two treatment plants (two sampling points per site). This was done to test the significance of increased chlorine contact time between the FE and DP. The distance between FE and DP at SWTP and KWTP are 23.3 and 7.1 m, respectively. The samples were collected in sterile 1.7 L Nalgene bottles containing 1 % sodium thiosulfate to dechlorinate the samples and transported in cooler boxes to the Applied and Environmental Microbiology Research Group (AEMREG) Laboratory, University of Fort Hare, Alice.

### Concentration of Viruses in Wastewater Samples and Extraction of Viral Nucleic Acids

The adsorption–elution method previously described by Haramoto et al. (2005) was used to concentrate the viral particles in the effluent samples with slight modifications. Samples were firstly pre-filtered using glass fibre (Millipore, Ireland) to remove debris and reduce clogging of filter membranes. Briefly, 5 mL aliquot of 250 mM AlCl_3_ was passed through an HA filter (0.45 µm pore size and 47 mm diameter; Millipore Ireland) to form a cation (Al^3+^)-coated filter, this was followed by filtering 1 L of the pre-filtered samples through the filter. Afterwards, 200 mL of 0.5 mM H_2_SO_4_ (pH 3.0) was passed through the filter to remove Al^3+^, and the viruses were eluted with 10 mL of 1 mM NaOH (pH 10. 8). The eluate was carefully recovered in a tube containing 50 µL of 100 mM of H_2_SO_4_ (pH 1.0) and 100 µL of 100 × Tris–EDTA (TE) buffer for neutralisation before further concentration using Centriprep YM-50 ultrafiltration device (Millipore) to obtain a final volume of approximately 700 µL. Each final concentrated sample was aliquoted in 200 µL and stored at −80 °C until ready to use. Storing viruses at temperature below −60 °C has been shown to result in insignificant loss of both titre and infectivity for periods longer than a decade (Gould 1999).

HAdV DNA was extracted from 200 µL of the concentrated samples using DNA extraction kits (Quick-*gDNA™* MiniPrep; Zymo Research, USA), following the manufacturer’s instruction. Purified viral DNA was eluted in 60 µL of DNA elution buffer. Extraction of the RNA viruses (HAV and RV) was done using RNA purification kits (Quick-RNA™ MiniPrep; Zymo Research, Irvine, USA). 100 µL of the concentrated samples was extracted and eluted in a final volume of 10 µL elution buffer as instructed by the manufacturer.

### Reverse Transcription of HAV and RV Genomes

The eluted 10 µL RNA genomes were converted into complementary DNA (cDNA) in a reverse-transcription step. The reverse-transcription step included a 20 µL (final volume) consisting of 10 µL RNA template, 1 µL of 100 µM Random Hexamer primer, 1 µL dNTP mix (10 mM each of GTP, ATP, CTP and TTP stock), 2.5 µL DEPC-treated water, 4 µL of 5 × RT buffer, 0.5 µL Ribolock RNase inhibitor and 1 µL of 200-U/µL RevertAid™ Premium reverse transcriptase (Fermentas, Burlington, ON, Canada). The mixture was briefly vortexed and centrifuged, and the transcription was carried out in a Dri-Block DB.2A (Techne, SA) at 25 °C for 10 min followed by incubation at 60 °C for 30 min and final incubation at 85 °C for 5 min. For RV, the RNA was initially subjected to denaturation for 5 min at 95 °C and flash chilling in ice for 2 min to separate the double-stranded of RV prior to the reverse transcription as previously described by Jothikumar et al. (2009).

### Construction of Standard Curves and qPCR Sensitivity Studies

Standard curves were plotted following the descriptions of Haramoto et al. (2008). For HAdV, viral nucleic acid was extracted from ATCC VR-6 (Strain Tonsil 99) reference strain using DNA extraction kits (Quick-*gDNA™* MiniPrep; Zymo Research, USA) while transcribed cDNAs from ATCC VR-1357 (Strain PA21) and ATCC VR-2274 (Strain 248) were used to construct the standard curves for HAV and RV, respectively. The extracted DNA/cDNA were quantified using a Qubit fluorometer (Invitrogen) followed by tenfold serial dilutions using nuclease-free water. The DNA/cDNA extracts from the samples and the positive control strains were subjected to qPCR simultaneously, each in triplicate. As previously described by Simmons and Xagoraraki (2011), the amplification efficiency and the detection limits of the qPCR assays were established and validated before their application to the sample extracts. The sensitivity and specificity of the assays were established using nucleic acid from stock cultures of HAV, RV and HAdV DNA from seven-fold serial dilution of the genomic extracts, while a detection limit of 10 copies of target DNA per reaction was set for each qPCR assays.

### TaqMan Probe-Based qPCR Assays for the Detection and Quantification of HAdVs, HAV and RV Genomes

TaqMan-based real-time PCR (qPCR) assays were used to determine the concentrations of viruses in the extracted samples in a StepOnePlus System (OPTIPLEX 755, Applied Biosystems). The amplification and real-time quantification of HAdV genomes in the samples was done by amplifying the hexon gene of the virus (Xagoraraki et al. 2007) while RV detection and quantification was done by amplifying the inner capsid protein VP6 as described by Lai et al. (2005). The qPCR was done in a 96-well plate by adding 5 µL of the HAdV DNA extracts/transcribed cDNA of HAV/RV to 20 µL of PCR “cocktail” mixture (12.5 µL of 2 × TaqMan universal PCR master mix, consisting of 0.05 u/µL Taq DNA polymerase, reaction buffer, 4 mM MgCl_2_ and 0.4 mM of each of dNTP; 400 nM forward primer; 400 nM reverse primer, 250 nM TaqMan probe and PCR grade water) in each well of the plate to make a final volume of 25 µL per reaction (Haramoto et al. 2008). All qPCR protocols were run for 45 cycles, and florescence activity data were collected at the end of each PCR cycle. This was followed by SDS software (Applied Biosystems) analysis to obtain quantitative data on the concentration of viral DNA in each well. Samples’ positivity was defined by a threshold cycle (*C*_T_) value of ≤35 while the limit of detection was demonstrated to be less than 10 viral genome copies per reaction. The primer sets and probes as well as the qPCR protocols used for the detection and quantification of the viruses are given in Table 1.

**Table 1:** Specific oligonucleotide primers and probes for the qPCR detection and quantification viral genomes

Enteric virus	Primer sequence (5′ → 3′) and TaqMan probe label	Reaction conditions (°C)	Reference	Control strain
Adenovirus	JTVX (F): 5′-GGACGCCTCGGAGTACCTGAG-3′	95°, 95°, 55°, 72°	Xagoraraki et al. (2007)	ATCC VR-6
	JTVX (R): 5′-ACIGTGGGGGTTTCTGAACTTGTT-3′	15′, 10″, 30″, 20″		
	JTVX (P): 5′-FAM-CTGGTGCAGTTCGCCCGTGCCA-BHQ-3′			
Hepatitis A Virus	HAV68 (F): 5′-TCACCGCCGTTTGCCTAG-3′	95°, 95°, 60°, 70°	Pinto et al. (2009)	ATCC VR-1357; Strain PA21
	HAV240 (R): 5′-GGAGAGCCCTGGAAGAAAG-3′	10′, 15″, 1′, 1′		
	HAV150 (P): 5′-FAM-CCTGAACCTGCAGGAATTAA-MGBNFQ-3′			
Rotavirus	JVK (F): 5′-CGATGGTTGATGCTCAAGATGGA-3′	95°, 95°, 55°, 72°	Jothikumar et al. (2009)	ATCC VR-2274; Strain 248
	JVK (R): 5′-TCATTGTAATCATATTGAATACCCA-3′	15′, 15″, 30″, 30″		
	JVK (P): 5′-FAM-ACAACTGCAGCTTCAAAAGAAGWGT-BHQ-3′			

*F* forward/sense, *R* reverse/antisense, *p* probe, *FAM* 6-carboxyfluorescein (reporter dye), *MGBNFQ* minor groove binder/nonfluorescent quencher, *TAMRA* 6-carboxy-tetramethylrhodamine (quencher dye), *BHQ* black hole quencher

### Characterisation of Human Adenovirus Species and Serotypes

Samples that were positive for HAdV from the qPCR were further subjected to qualitative PCR to detect the epidemiologically important adenovirus species and serotypes. Adenovirus species and serotypes assayed for included Ad40 and Ad41 (belonging to species F), Ad3, Ad7 and Ad21 (belonging to species B), Ad1, Ad2, Ad5 and Ad6 (belonging to species C) and Ad4 (belonging to species E) (van Heerden et al. 2005; Jiang 2006; Sibanda and Okoh 2012). Serotype-specific PCR assays as described by Metzgar et al. (2005) were used to detect the various serotypes with some modifications. The PCR assays consisted of 5 µL of viral DNA added to 20 µL of reaction buffer (12.5 µL of 2 × PCR master mix, 0.5 µL each of forward and reverse primers and 6.5 µL of nuclease-free water) to make a final volume of 25 µL per reaction. The primer combinations and the molecular weight (in base pairs) used for the detection of the various species and serotypes are shown in Table 2. The amplicons were resolved on 1.5 % agarose gel electrophoresis stained with ethidium bromide in TBE (Tris–borate-EDTA) buffer at 100 V for 1 h. The resolved amplicons were visualised and digitised using trans-illuminator (BioDoc-It System; UVP Upland, CA 91786, USA). Table 3 lists the adenovirus ATCC reference strains used in the study for the detection of HAdV species and serotypes.

**Table 2:** Primer sets and amplicon sizes for the detection of HAdV species and serotypes

Species	Serotypes	Primer	Sequence (5′–3′)	Amplicon size (bp)	Target region	References
B	Ad3	Ad3F	GGTAGAGATGCTGTTGCAGGA	503	Ad3 hexon	Metzgar et al. (2005)
		Ad3R	CCCATCCATTAGTGTCATCGGT			
	Ad7	Ad7F	GGAAAGACATTACTGCAGACA	311	Ad7 hexon	
		Ad7R	AATTTCAGGCGAAAAAGCGTCA			
	Ad21	Ad21F	GAAATTACAGACGGCGAAGCC	237	Ad21 hexon	
		Ad21R	AACCTGCTGGTTTTGCGGTTG			
C		AdCF	TGCTTGCGCTHAAAATGGGCA		AdC fibre	Adhikary et al. (2004)
	Ad1	Ad1R	CGAGTATAAGACGCCTATTTACA	630	Ad1 fibre	
	Ad2	Ad2R	CGCTAAGAGCGCCGCTAGTA	204	Ad2 fibre	
	Ad5	Ad5R	ATGCAAAGGAGCCCCGTAC	455	Ad5 fibre	
	Ad6	Ad6R	CTTGCAGTCTTTATCTGAAGCA	929	Ad6 fibre	
E	Ad4	Adeno4.U3	CAAGGACTACCAGGCCGTCA	254	4 hexon	Hough et al. (2002)
		Adeno4.L1	TTAGCATAGAGCATGTTCTGGC			
F		AdF1	ACTTAATGCTGACACGGGCAC		AdF fibre	Xu et al. (2000)
	Ad40	K402	CACTTAATGCTGACACG	152	Long fibre gene	
	Ad41	K403	ACTGGATAGAGCTAGCG

**Table 3:** Viral control strains used for HAdV species and serotype detection

Adenovirus serotype	Reference number	Strain
Adenovirus T 21	ATCC(R) VR-256	Strain AV 1645
Human adenovirus 1	ATCC VR-1	Strain Adenoid 71
Human adenovirus 2	ATCC VR-846	Strain Adenoid 6
Human adenovirus 3	ATCC VR-3	Strain GB
Human adenovirus 40	ATCC VR-931	Strain Dugan
Human adenovirus 40	ATCC VR-1572	Strain R1-67
Human adenovirus 5	ATCC VR-1516	Type 5 Reference Material
Human adenovirus 6	ATCC VR-6	Strain Tonsil 99
Human adenovirus 7	ATCC VR-7	Strain Gomen
Human adenovirus 41	ATCC VR-930	Strain Tak (73-3544)

### Quality Control

Positive controls (spiked samples of known viral DNA/cDNA concentrations) and negative controls (nuclease-free water and PCR buffer) were included in all PCR assays. Also, in order to ascertain the efficiencies of the sample concentrations steps, nucleic acid extractions, primer combinations and the qPCR assays, and to avoid false-positive/false-negative results, initial testing of these processes were carried out by spiking known amount of the control (reference) viruses into sterile distilled water and some test wastewater effluent samples. These were taken through the whole processes and were successfully amplified using the specific primer combinations. Due to extreme sensitive nature of qPCR, cross contamination of samples and amplified product were eliminated by carrying out DNA extraction and PCR assays in separate rooms. DNAZap™ (Ambion^®^) solution was always used to wash micropipettes before every PCR assay to completely degrade all DNA and RNA which might contaminate our samples, while pre-sterilised filtered racked micropipette tips were used throughout the assays.

### Detection and Enumeration of Faecal Coliforms

Faecal coliforms densities were enumerated by membrane filtration techniques according to standard methods (APHA, 1998). Appropriate serial dilutions of each of the samples were made and 100 ml from each of the dilutions was filtered through membrane filters (47-mm diameter, 0.45 µm pore size; Pall Corporation, Ann Arbor, Michigan) with the aid of a vacuum pump. The membrane filters were placed on m-FC agar (Merck, Wadeville, South Africa) and incubated at 44.5 °C for 24 h. Colonies that exhibit various shades of dark blue were counted and reported as CFU/100 ml of wastewater sample analysed.

### Statistical Analysis

Calculation of means and standard deviations were performed using One-way ANOVA (SPSS 22.0 version for Windows program). Comparison of differences in means between paired samples was done using the Paired-Samples *T* test. The correlation between FC and HAdV concentrations was determined by Linear Regression analysis using HAdV concentration as dependent variable and FC count as the predictor at *P* values equal to 0.05.

## Result

### Real-Time PCR Sensitivity, Specificity and Detection Limits

The sensitivity and specificity of the primers and probes used for the qPCR assays were validated using the respective viral nucleic acid extracts as templates. The control viral strains of HAdV (ATCC VR-6), HAV (ATCC VR-1357; strain PA 21) and RV (ATCC VR-2274; strain 248) were all detected by qPCR. The resultant standard curves (HAdV, slope-3.53, Y-intercept 28.34; HAV, -2.94, Y-intercept 33.11 and RV, slope-3.95, Y-intercept 38.94) showed strong correlation coefficients (*R* ^2^) of 0.99 (for HAdV and HAV) and 0.97 (for RV), respectively. Amplification efficiencies were >92 % for all reactions, while no amplification was observed in the negative controls.

### Detection and Quantification of Human Adenovirus Genome

**Fig. 1:**
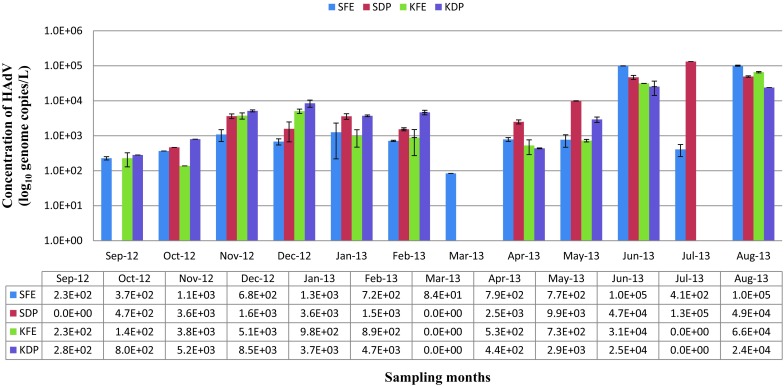
Monthly variation in HAdV concentrations in wastewater samples from the two study sites. *Key* SFE-SWTP final effluent sample; SDP-SWTP discharge point sample; KFE: KWTP final effluent sample; KDP: KWTP discharge point sample

The distributions and concentrations of HAdV varied widely at all sampling points. Figure 1 summarises the results for HAdV detection in the effluent samples. HAdV was detected at all sampling points, and in 62.5 % (30/48) of the samples tested by qPCR with, concentrations generally ranging between 8.4 × 10^1^ and 1.3 × 10^5^ genome copies/L. About 53 % (16/30) of the HAdV-positive samples were from SWTP (9 positive samples from SWTP final effluent (SFE) with concentration ranging between 8.4 × 10^1^ and 1.3 × 10^5^ genome copies/L and 7 positive samples from SWTP discharge point (SDP) with concentrations ranging between 4.7 × 10^2^ and 5.0 × 10^4^ genome copies/L), while 46.7 % (14/30) of the HAdV-positive samples were from KWTP (7 positive samples from KWTP final effluent (KFE) with concentrations ranging between 2.3 × 10^2^ and 6.6 × 10^4^ genome copies/L and 7 positive samples from KWTP discharge point (KDP) ranging between 2.8 × 10^2^ and 2.4 × 10^4^ genome copies/L).

**Fig. 2:**
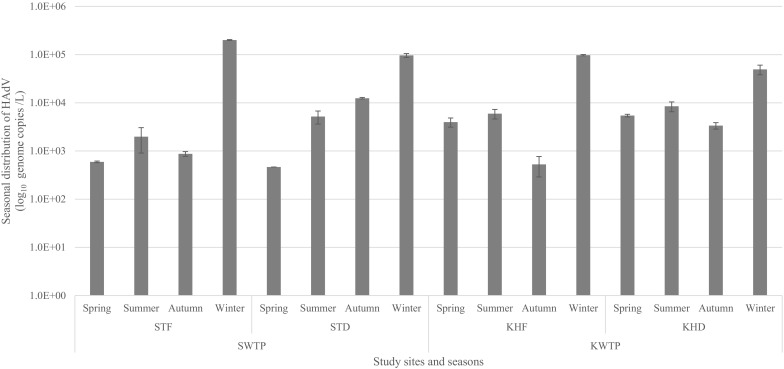
Seasonal distribution of HAdV concentration at all sampling points

At SWTP, the lowest concentration of HAdV (84 genome copies/L) was recorded in March 2013 and the highest concentration (1.3 × 10^5^ genome copies/L) in August 2013. At KWTP, the lowest concentration (1.3 × 10^2^ genome copies/L) of HAdV was observed in October 2012 while the highest concentration (6.5 × 10^4^ genome copies/L) was in August 2013. No consistent seasonal trend was observed in the distribution of HAdV at both study sites over the sampling period. However, increased concentrations of HAdV were generally observed in the winter months at all sampling points (Fig. 2).

### Characterisation of Human Adenovirus Virus Species and Serotypes

Molecular typing of the HAdV-positive samples by PCR revealed the prevalence of species B (serotype Ad3) with a detection rate of 86.7 % (26/30 of HAdV-positive samples), while species F (serotype Ad41) was detected in 6.7 % (2/30) of the samples as shown in Table 4. Other species and serotypes tested for were not detected in the effluent samples.

**Table 4:** Characterisation of HAdV in the effluent samples into species and serotypes

Study site		Sampling month											
		Sept-12	Oct-12	Nov-12	Dec-12	Jan-13	Feb-13	Mar-13	Apr-13	May-13	Jun-13	Jul-13	Aug-13
SWTP	STF	AdB	AdB	−	−	+	AdB	AdB	+	−	AdB	AdB	AdB
	STD	−	AdB	−	AdB, AdF	AdB	−	−	AdB	AdB	AdB	−	AdB
KWTP	KHF	AdB	−	AdB	+	−	AdB	−	AdB, AdF	−	AdB	−	AdB
	KHD	AdB	−	+	AdB	−	−	−	AdB	AdB	AdB	−	AdB

*AdB* human adenovirus serotype 3, *AdF* human adenovirus serotype 41; *+HAdV* detected by qPCR, *−HAdV* not detected by qPCR

### Detection and Quantification of Hepatitis A Virus and Rotavirus

The detection of HAV and RV in the samples was insignificant. RV was not detected in any of the samples throughout the sampling period while HAV was only detected in 3 (6.25 %) of the samples, but, at concentration <1 genome copies/L, which was far below the detection limit set for viral genome amplification by the qPCR.

### Detection and Enumeration of Faecal Coliforms

**Fig. 3:**
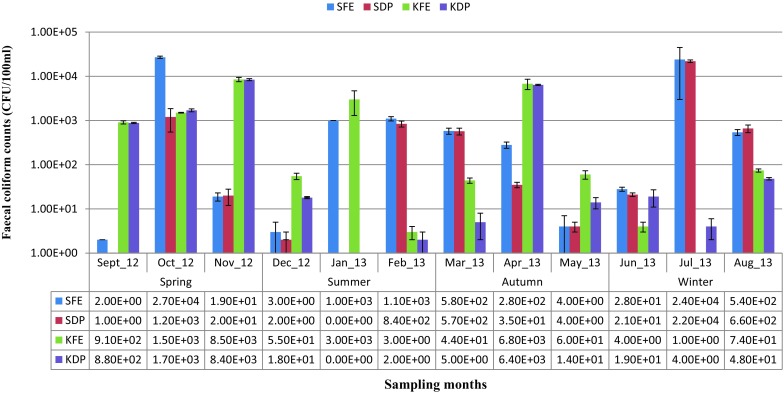
Monthly variation in FC counts in wastewater samples from the two study sites. *Key* SFE-SWTP final effluent sample; SDP-SWTP discharge point sample; KFE: KWTP final effluent sample; KDP: KWTP discharge point sample

Similar to the observation for HAdV, the detection and distribution of FC in the effluent samples also varied widely. About 96 % (46/48) of the effluent samples were positive for FC at counts ranging from 1 CFU/100 ml to 2.7 × 10^4^ CFU/100 ml. The FC counts in the samples generally ranged at both sites as follows: SWTP (1 − 2.7 × 10^4^ CFU/100 ml) and KWTP (1 − 8.5 × 10^3^ CFU/100 ml). The counts also varied significantly (*P* < 0.05) among the seasons with the highest FC counts at SWTP (2.7 × 10^4^ CFU/100 ml) and at KWTP (8.5 × 10^3^ CFU/100 ml) both occurring in the spring months as shown in Fig. 3, while the lowest counts were observed in summer (January 2013) also at both sites. There appears to be a similar trend in the counts of FC at both treatment plants with the highest and the lowest FC counts occurring at spring and summer, respectively. Of the effluent samples collected at SWTP, 25 % (6/24) had FC counts above the 1000 CFU/100 ml for discharged final effluents as recommended by the Department of Water Affairs and Forestry, Republic of South Africa (DWAF 1999) while 29.2 % (7/24) were above this recommended limit at KWTP.

## Discussion

South Africa is currently experiencing an epic drought, a situation which has led to the declaration of 5 out of its 9 provinces as drought disaster areas, and this condition is seriously threatening food security in the country. Increasing awareness regarding the need for efficient use of water resources has placed emphasis on wastewater recycling as an important source of replenishing freshwater supply in South Africa (Dungeni et al. 2010). However, different studies have revealed the contributions of inadequately treated effluents to the pollution of freshwater resources which sometimes directly serve as sources of water for many rural dwellers still lacking access to improved water sources in the country (Mema 2009; Council for Scientific and Industrial Research CSIR 2010; Naidoo and Olaniran 2014). Inefficient wastewater treatment process will lead to the discharge of final effluents with unacceptable microbiological quality into receiving watersheds with associated public health risk (Casadio et al. 2010).

Human adenoviruses are commonly found in environments where human faecal and sewage contaminations have occurred and have been implicated as causative agents of persistent infections and outbreaks in drinking and recreational waters (World Health Organisation, WHO 2005). They have also been shown to be resistant to tertiary wastewater treatments and UV radiation, as well as chemical, physical and adverse pH conditions, which allows them to survive for a prolonged period in the environment (WHO, 2005; Carducci and Verani 2013). Proper monitoring of human enteric viruses in water system is very important, different reports have suggested that viral agents are the causative agents of approximately 50 % of all known gastrointestinal infections (Centers for Disease Control and Prevention CDC 1988; Choi and Jiang 2005). In this study, 62.5 % (30/48) of the wastewater effluent samples analysed tested positive for HAdV, with no consistent seasonal variations, however, adenovirus concentration generally increased in winter months (June, July, August) at all sampling points. The increased concentration observed in winter months may be due to favourable environmental conditions such as lower temperature compared to other seasons of the year. Lipp et al. (2001) reported that aquatic environment factors such as temperature, pH, ultraviolet light and salinity play an important role in the survival of microorganisms and at higher temperatures, inactivation of viruses occurs due to the denaturation of its protein and nucleic acids while lower temperatures support viral survival for longer periods. Higher incidence of HAdV in winter months has also been reported by Rigotto et al. (2010). Similarly, some studies have documented high prevalence of adenoviruses from different environmental matrices (including human faeces, raw and treated wastewater, beach water, river water, seawater, shellfishes), and have suggested their use as indices of faecal pollution from human sources because of their stability in the environment, host specificity, persistent infections and less or no variability in their seasonal occurrences (Vieira et al. 2012; Bofill-Mas et al. 2013).

As observed in the study, the treatment processes did not completely remove enteric viruses from the effluent samples collected from both treatment plants as HAdV were detected in the discharged effluents all year round even in effluents with adequate free chlorine concentrations. For instance, at SWTP, HAdV was detected at about 1.0 × 10^5^ genome copies/L (Fig. 1) in the SFE samples with free chlorine concentrations of 0.30 mg/L, whereas at SDP (point of discharge into receiving watershed), HAdV was detected at a concentration of 4.6 × 10^4^ genome copies/L with free chlorine concentration of 0.28 mg/L in June 2013. A similar observation was noted at KWTP, where HAdV detection at KFE was 3.1 × 10^4^ genome copies/L with effluent-free chlorine concentration of 0.37 and at the discharge point (KDP), HAdV detection in the effluent was 2.5 × 10^4^ genome copies/L with free chlorine concentration of 0.35 mg/L. Our observation is corroborated by Manios et al. (2006) and Carducci et al. (2008) who stated that standard treatment processes are insufficient to reduce viral load below risk level. The shielding effect associated with the adhesion of viral particles to particulate matter in treated wastewater may be responsible for the survival of the adenovirus strains detected in the study (Enriquez et al. 1995; Carter 2005). The finding of this study provides definite evidence of the occurrence and discharge of HAdV from wastewater treatment plants into the aquatic milieu of the Eastern Cape Province, South Africa, thus presenting a potential public health risk.

Even though, HAdV serotypes 40 and 41 have largely been reported as the most common aetiological agents of acute viral gastroenteritis in children throughout the world besides the group A rotavirus (Xagoraraki et al. 2007; Carraturo et al. 2008), our findings reveal adenovirus serotype 3 (species B) as the predominant HAdV serotype and was detected in 86.7 % (26/30) of the HAdV-positive samples, while serotype 41 (species F) was only detected in 6.7 % (2/30) of the positive samples, and 13.3 % (4/30) of the samples were not positive for any of the assayed species/serotypes. The high prevalence of HAdV B, as seen in this study could be an indicator of the predominant adenovirus species in circulation among the human population in our study area. Adenovirus serotype 3 together with other serotypes (including 5, 7 and 21) has been associated with adenoviral lower respiratory tract infection epidemic (LRTI) (WHO, 2005; Alharbi et al. 2012). Type 3 adenovirus has also been liked to outbreaks of conjunctivitis (Abelson and Shapiro 2010). However, contrary to our observation, Michigan, Fong et al. (2010) documented adenovirus type 3 as the least-detected serotype from raw and primary effluents in their report on the quantitative detection of adenovirus in some environmental waters and concluded that surface water impacted by discharged wastewater effluents may not be suitable for full-body recreational activities. Also, van Heerden et al. (2005) reported HAdV D as the predominant species detected while investigating the prevalence and typing of adenovirus species in some river and treated drinking water samples in South Africa. The detection of HAdV serotypes in the treated effluent suggests that the human population in the study area could have suffered adenovirus-related illness most importantly during the sampling period.

The detection of HAV and RV in the effluent samples was negligible over the sampling period, while RV was not detected at all in any of the samples, HAV was only detected in two samples (4.1 %) from SDP but at concentration <1 genome copies/L, below our set detection limit. The reliability of the results obtained in this study was ensured by putting in place adequate quality control measures including elimination of false-positive/false-negative amplifications, and running of assays in replicates. Nondetection of RV as observed in this study is similar to the finding of Hot et al. (2003) who reported 0 % detection of rotavirus in 68 surface water samples while investigating the detection of somatic phages, infectious enteroviruses and enterovirus genomes as indicators of human enteric viral pollution in four French rivers. A similar observation of nondetection of RV was also made by Symonds et al. (2009) while reporting of the detection of eukaryotic viruses in wastewater sample from the United States. The failure of RV detection may suggest that individuals in the study area were not shedding this potentially pathogenic virus at the time of the study or that the RV was completely inactivated by the disinfectant (chlorine) used at the treatment works since the viral genome were successfully amplified from the control strain. Nondetection of HAV as observed in this study is also comparable to the report of Prado et al. (2012) who did not detect HAV in 24 treated effluent samples over the course of 1-year sampling period or urban wastewater from Rio de Janeiro, Brazil. Although HAV might have been present in our effluent samples, however their concentrations might be far below the detection limits of 10 genome copies/L set for the study.

Although, faecal indicator bacteria may not be pathogenic, their presence in water systems often signify the likely presence of other faecal transmitted pathogens and reflects impairment in water quality with increased risk of gastrointestinal and other waterborne illnesses (Bhandaram et al. 2011; Sibanda et al. 2013). The effluents samples in this study mostly complied with recommended guidelines for FC counts (1000 CFU/100 mL) for larger parts of the sampling period. However, counts above this limit were observed about 27 % of the samples. The FC counts were not significantly (*P* < 0.05) different between the FE and the DP samples at both treatment plants as shown by the Paired-Samples *T* test analysis. This could attributed to the relative short distances (23.3 m at SWTP and 7.1 m at KWTP) between the final effluent tanks and the discharge points allowing little or no further disinfection action of the residual chlorine before discharging the effluents into the receiving watershed.

While this study showed higher prevalence of FC in spring than other seasons of the year, other studies have shown different prevalence and distributions of FC in some aquatic environment. In his report while working on distribution of presumptive FC around Rothera Point, Antarctic Peninsula, Hughes (2003) reported low concentration of FC in summer and suggested this might have resulted from the biological damaging effect of solar radiation in summer. He observed high concentration of FC in winter and suggested that this might be due to a combination of factors such as increased input by migrating wildlife, low solar radiation and sewage contribution. He concluded that environmental factors including solar radiation, water salinity, temperature, sea ice conditions and faecal input by human and local wildlife populations affect FC distribution. Wani et al. (2013) also documented a greater efficiency in the removal of faecal indicator bacteria including FC, *E. coli* and faecal streptococci in summer and autumn months, and least in winters while investigating the effect of seasonal change on the removal efficiency of a FAB (Fluidized Aerobic Bioreactor)-based sewage treatment plant and the impact of the discharged effluent in the vicinity of Dal Lake.

Contrary to the trend observed for FC counts, slight variations were observed for adenovirus concentrations between the FE and the DP samples. However, the concentrations of HAdV in the samples did not correlate (*P* < 0.05) with FC counts as observed by the linear regression analysis (data not shown). This observation is not surprising as several other studies have highlighted the lack of correlation that exists between faecal indicator bacteria and the presence of enteric viruses in water quality monitoring (Jiang et al. 2007; Ahmed et al. 2008; Hata et al. 2012). An important implication of this observed phenomenon will be that, while the discharged treated effluent complied with recommended guideline for faecal coliform for most part of the sampling period, they however, carry high loads of HAdV which represent health risk to persons coming in contact direct or indirectly with these effluents.

In conclusion, monitoring of discharged final effluents of wastewater treatment plants could serve as an important approach to ensure the protection of surface waters (which often serve as the receiving watershed) from the impact of poorly treated effluents, suggest the prevailing pathogen(s) circulating among the human population in a given area and help in making informed decisions to protect public health. The presence of high concentrations of HAdV in the discharged final effluents signifies the inefficiency of the treatment process to adequately remove the potential pathogen. This constitutes a significant public health risk particularly among immunocompromised persons given that a significant number of rural dwellers in the study area still depend on untreated surface water for various domestic and agricultural uses. As demonstrated in this study and other related studies, real-time PCR is an important and a powerful tool for rapid detection and quantification of viral nucleic acid in environmental samples. However, due to its inability to discriminate between infectious and non-infectious viral particles, it is imperative to carry out virus infectivity assays using appropriate techniques to ascertain the infectivity capabilities of the viral particles detected by the qPCR.
